# Investigation of Cognitive Impairment in the Course of Post-COVID Syndrome

**DOI:** 10.3390/diagnostics13162703

**Published:** 2023-08-18

**Authors:** Milena Dimitrova, Yoanna Marinova, Dancho Dilkov

**Affiliations:** Psychiatry Clinic, Military Medical Academy, 1606 Sofia, Bulgaria

**Keywords:** post-COVID syndrome, cognitive impairment, cognitive disturbance, memory, attention deficit, mild cognitive deficit, aphasia, pro-cognitive agent, neuroprotective agent, COVID-19

## Abstract

(1) Background: The study presents results from an investigation of cognitive impairment in patients hospitalized in the first psychiatric clinic in Bulgaria to treat patients with COVID-19 during the pandemic period between 2020 and 2022. One hundred and twenty patients who had recovered from acute COVID-19 infection (up to 12 weeks ago) and had no previous history of cognitive impairment participated in the study. In 23 of them (19.17%), disturbance of cognitive functioning was observed. (2) Methods: All 23 patients underwent neuropsychological (Luria’s test, Platonov’s Maze test, MMSE, Boston Naming test) and neuroimaging examinations. Only seven of them had evidence of cortical atrophy on CT/MRI images. The most significantly demonstrative image of one of those patients is presented. (3) Results: The neuropsychological testing results of both groups show a certain decrease in fixation and memory retention as well as in the range, concentration, distribution and switching of attention. Deviations from the norm on the MMSE, as well as on the Boston Naming Test, were found in the group of patients with cortical atrophy (mild to moderate aphasia). Neuroprotective agents such as Citicoline, Piracetam and Memantine were prescribed to the patients with evident cortical atrophy. After 3 months, positive results of the neuropsychological examination were reported in both groups. (4) Conclusions: Although there are limited data on the benefit of prescribing pro-cognitive agents in the post-COVID period, our clinical experience suggests that it might be useful in the recovery process from the infection’s consequences on cognition for patients with brain pathology.

## 1. Introduction

In December 2019, Wuhan, the capital of Hubei Province, became the center of a new outbreak of pneumonia of unknown cause. By 7 January 2020, Chinese scientists had isolated the new coronavirus from patients with pneumonia caused by it. The virus, also known as SARS-CoV-2, was the cause of the Severe Acute Respiratory Syndrome. The disease was later named COVID-19 by the World Health Organization in February 2020 [1,2,3]. In the beginning of March 2020, WHO officially defined the COVID-19 pandemic and since then, numerous questions have been raised in terms of scientific research on a global scale.

SARS-CoV-2 could affect multiple organs and tissues, including the central nervous system. Manifestations of the disease observed after the acute stages are relatively common and are united under the general term and the novel clinical entity called “post-COVID syndrome” or “prolonged-COVID-19” [4,5]. Acute COVID-19 usually lasts up to 4 weeks from the onset of the first symptoms of the disease. Typically, no replication-competent SARS-CoV-2 viral particles are isolated after this period. “Post-COVID syndrome” summarizes the residual health consequences, and it is defined as a combination of persistent symptoms and delayed or long-term complications after the acute phase [5].

The nature of the clinical complications caused by COVID-19 is heterogenous and can affect multiple organs, not only the respiratory system. Nalbandian et al. summarize the most common symptoms after acute COVID-19 infection. Fatigue, muscle weakness and joint pain are symptoms affecting the biotonus and the quality of general functioning. Typical post-COVID symptoms are also palpitations, chest pain, increased coagulation and chronic kidney damage. Most common psychiatric and neuropsychiatric symptoms include anxiety, depression, sleep disorders, post-traumatic stress disorder, headache and cognitive disorders (brain fog) [5].

Cognitive disturbances are among the most common neuropsychiatric symptoms reported by patients after the acute COVID-19 phase [6,7]. Cognitive impairment is a broad term used to describe a variety of different brain functions. Evidence regarding the prevalence, characteristics and mechanisms associated with the cognitive dysfunctions following COVID-19 is still scarce. Mixed results have been found in several cognitive domains, particularly attention, executive functions, and episodic memory [8,9,10,11]. Zhu et al. found cognitive impairment may be related to the underlying inflammatory processes in patients who recovered from the infection. The severity of cognitive disturbances correlates to the level of inflammation and, more specifically, to the CRP levels of COVID-19 survivors [9]. Moreover, evident relationship between the severity of the cognitive impairment and the D-dimer levels during the acute infection and the pulmonary post-COVID disfunction was found [12]. Hypoxia and the need for oxygen therapy are other factors that lead to an increasing risk of discrete brain damage. This might bring further attention to the patients with moderate to severe COVID-19 infection.

However, other studies provide evidence that cognitive impairment might be found in patients with mild forms of COVID-19 as well. The consequences on the cognition after COVID-19 have to be suspected even in patients without any other serious medical complications. Researchers found a sustainable probability that COVID-19 might cause cognitive impairment, despite the clinical severity of the infection. Zhao et al. investigated a wide range of cognitive functions in patients with asymptomatic to moderate COVID-19. They found specific chronic cognitive changes presented with significant decline in episodic memory up to 6 months after infection and even greater decline in vigilance with time on task—up to 9 months [13]. This is also supported by Del Bruto et al., who measured 18,1 times higher risk for developing cognitive decline among patients with mild symptomatic COVID-19 infection in a study published in 2021 [14].

Clarification of the affected specific cognitive domains and targeting their corresponding brain circuits might improve the cognitive recovering processes of these patients. Several studies already show promising data on the topic. Ladopoulos et al. investigated brain structural changes in COVID-19 survivors through neuroimaging examinations and found that medial temporal structures and hippocampus are affected. Therefore, deficits of memory and other cognitive domains might be expected, as well as short-term memory disfunction as a consequence of the abnormal consolidation of information [15]. Hosp et al. found changes in metabolism in the frontoparietal lobe in the subacute stage of COVID-19, leading to the conclusion of attentional network impairment [16].

Other studies on COVID-19′s cognitive disturbances clarify the multilateral nature of the affected cognitive domains. Global cognitive impairment after COVID-19 consists of changes in memory, attention, executive functions, and verbal fluency [11]. A study published in 2022 investigated the patterns and prevalence of cognitive impairments in patients with prior acute COVID-19 infection. It was observed that memory, attention and executive functions are the most affected cognitive domains. More specifically, memory disturbance was presented by the delay of recall and learning. Abstraction, inhibition, set shifting and focus, and selectivity of attention were the most commonly impaired executive functions. Language and visuospatial functions were less and rarely affected [17]. A study with 29 patients from 2021 conducted in Copenhagen investigated the frequency, pattern and severity of cognitive impairments 3–4 months after COVID-19 hospital discharge. They found clinically significant cognitive impairment in 59–65% of the patients, with verbal learning and executive functions being mostly affected. A systematic review published in 2022 on COVID-19-associated cognitive impairment concluded the most affected cognitive domains were attention, episodic memory, processing speed and executive functions [18]. A systematic review and meta-analysis with 1,285,407 participants from 32 countries on the long term COVID-19-associated physical and mental sequelae found cognitive deficits in 19.7% and memory impairment in 17.5% of them [19]. Another study from 2022 with 63 patients with subjective cognitive complaints after COVID-19 infection showed results of impact on attention, learning and long-term memory. Sixty two percent of the subjects showed attention deficit, which was the most common cognitive impairment, followed by executive functioning affecting 43% of the sample. Moreover, a significant correlation between executive functioning and attention was found, suggesting a codependent relationship between these two domains [20].

Cognitive assessment of patients with recent COVID-19 infection might be useful to establish the level of the cognitive impairment in order to take timely measures to prevent the chronic course of these complications.

The aim of the study is to present the results from our investigation of cognitive impairment in patients who overcame acute COVID-19 infection and have persistent complaints in cognitive functioning during the post-COVID period (up to 12 weeks after the first symptoms of acute infection).

## 2. Materials and Methods

Initially, 120 patients aged between 25 and 71 years were investigated. They declared symptoms (fever of at least 38 °C, cough, myalgia, tiredness, loss of smell and taste) of an acute COVID-19 infection (up to 12 weeks before entering the study) and complained of subjective cognitive decline in the post-COVID period. They have no prior history of cognitive impairment.

The inclusion criteria for this study were as follows:

(1) history of COVID-19 symptoms and positive PCR of serology (anti-SARS-CoV-2 IgM or IgG in the past 12 weeks;
(2) subjective cognitive complaints;
(3) minimum age of 18 years.

Evidence of medical history of neurological or psychiatric disorder before the infection was the exclusion criterion.

After reviewing the medical records, 26 of the participants were excluded as probable COVID (negative results when tested for SARS-CoV-2 or not tested at all). Thirty-seven of the remaining participants had documented anamnesis of neurological or psychiatric condition. The final sample included 57 participants. All of them went under neuropsychological testing and objective cognitive decline was found in 23 of them.

A comprehensive neuropsychological testing was conducted by applying standardized methods for each of the following cognitive domains: general cognitive assessment, attention and processing speed, short- and long-term memory, vocabulary and naming abilities. A general cognitive assessment was conducted by MMSE (Mini Mental State Examination). A. Luria’s “10 words” test was used for memory evaluation. Bourdon’s Letter correction test and Platonov’s Maze test/interlaced lines/were applied for attention and processing speed evaluation. The Boston Naming test by Kaplan and Goodglass diagnosed aphasia (it is a wide-range naming vocabulary test consisting of 60 pictures, ordered from the easiest to the most difficult level).

The Mini-Mental State Examination or MMSE, created by Folstein in 1975, is a simple screening test evaluating general cognitive functioning [21]. MMSE is applicable and very useful in the clinical practice. It includes items questioning orientation for time and place, focus and concentration, attention, memory, naming of objects, executive functioning and visuospatial skills. Its maximum score is 30 and results below 25 are equal to mild cognitive deficit.

Luria’s Memory Word Test was created by Luria in 1962, and it was improved and systematized by Christensen in 1975 [22,23]. It is a brief and easily administered test that is used to assess the qualitative and quantitative aspects of verbal learning and memory processes. The test itself is a task consisting of 10 words, read by an interviewer with a one-second interval between each word, in ten consecutive trials. The words are pronounced in a particular order throughout the trial. The interviewer repeats the instructions before each trial. After 30 min, the subject repeats the test for one trial only. The time required for administration is 10 to 15 min [24].

The Bourdon Letter correction test/Bourdon Alphabet Test/evaluates the level of attention and stability of concentration in the investigated subjects. It consists of 40 lines of letters and each line has 40 letters. The task is to sequentially search for and select certain characters in each line, starting from the top row from left to right. The test is normally conducted within 5 to 10 min

Platonov’s interlaced lines or maze test is a five-to-seven-minute-long pen-paper task that measures the attention and planning ability of the respondents. The task consists of mental tracing of interlaced lines from their beginning point to their end and the responder has to number each end of the lines with the number of its corresponding beginning point. The completion of the task also integrates working memory, decision making and cognitive flexibility. The average level of attention span is equal to 8–10 correct answers in up to 120 s [25].

The Boston Naming test by Goodglass and Kaplan created in 1983 is a popular quantitative measure of assessing naming ability. It consists of 60 pictures ordered from the easiest to the most difficult level. The time for answering is 20 s for each picture and the interviewer records each answer and the latency period for answering. The test also evaluates language skills based on perceptual modalities (auditory, visual, and gestural), processing functions (comprehension, analysis, problem-solving) and response modalities (writing, articulation, and manipulation) since the interviewer is allowed to give cues from different modalities if the subject experiences difficulties with completing the task. The score is measured in percentiles (0–100%) and each given answer or cue has meaning in forming the final result [26].

The MRI apparatus used in the investigation is the Magnetom Harmony 1T model by Siemens, Munich, Germany (2000). The CT scanner used in the survey are spiral scanner Somatom Plus 4 by Siemens (2000) and multislice spiral scanner Light Speeed 16 by General Electric, Boston, MA, USA (2004).

**Scheme 1:**
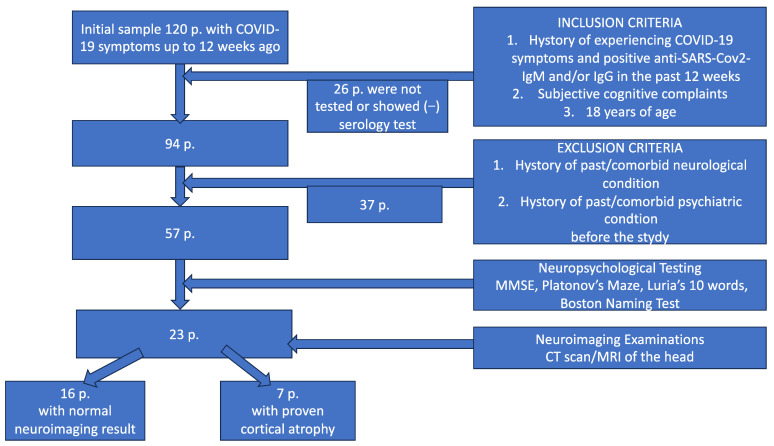
Flow diagram illustrating the methodology of the study (inclusion and exclusion criteria, neuropsychology testing and neuroimaging examinations). CT—computed tomography; MRI—magnetic resonance imaging.

The methodology of the study with its inclusion and exclusion criteria as well as the initial and final sample of the participants of the study are shown in Scheme 1.

## 3. Results

SARS-CoV-2 is a virus with proven neurotropic properties. The neuropsychiatric manifestations associated with COVID-19 are consequent from the penetration of the virus particles into the central nervous system, and depending on the severity of the illness, cognitive processes are impaired to a certain extent. These scientific findings about the viral pathophysiological mechanisms also enabled the timely recognition of cognitive disturbances, which are among the most common neuropsychiatric symptoms reported by patients after the acute phase of the disease [27].

After applying the exclusion criteria, from the group of 120 patients remained a final sample of 57 participants. All of them went under neuropsychological testing and objective cognitive decline was found in 23 (19.17%; 12 men and 11 women, aged 25–71 with mean age of 48 years SD 4.6). All of the 23 patients underwent neuroimaging examinations of the head as well. Only seven of them appeared with evidence of cortical atrophy, while the rest had normal MRI/CT scan findings.

The group of seven patients with proven cortical atrophy were aged between 55 and 71 years, mean age 61, SD 6.9. Seventy-one percent (five) of them were males and the rest (two) were females, 85% were married and 71% had graduated from university. They have been diagnosed by a psychiatrist with a comorbid psychiatric disorder such as general anxiety disorder, bipolar affective disorder, major depressive disorder, organic delusional disorder, and benzodiazepine and substance abuse syndrome (alcohol). The patients from this group did not have a history of previous psychiatric disorder and all of the comorbid psychiatric diagnoses were assessed after the COVID-19 infection. Hypothetically, they are related to the disease and the inflammatory processes or they are a result of the individual’s traumatic experience. The history of passing an acute COVID-19 infection in this group of patients is very likely to have increased the perception of cognitive disturbances during the post-COVID period.

The results of the neuropsychological examinations indicate that there is a slight to moderate decrease in fixation and memory retention as well as a moderate decrease in the range, concentration, distribution and switching of attention. The MMSE scores for general cognitive assessment were in the lower end of the norm, borderline with scores of mild cognitive deficit. Mild to moderate aphasia was objectively determined with the Boston Naming test.

**Figure 1:**
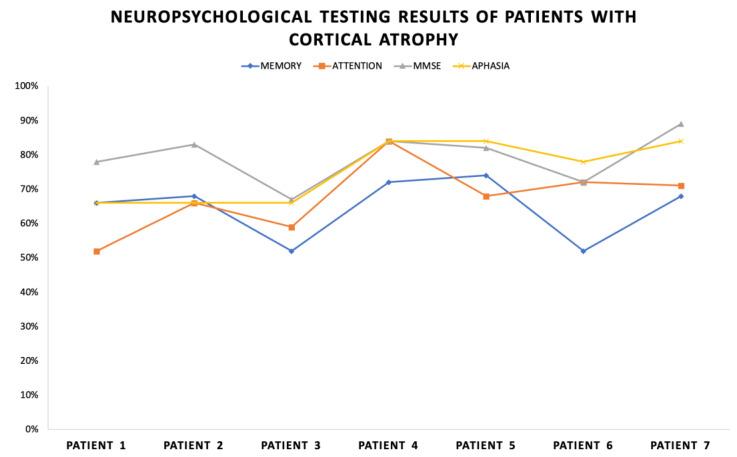
Neuropsychological testing results of patients with cortical atrophy.

Figure 1 shows the results of each patient in this group according to the different tools of the used neuropsychological battery. The largest decline in the cognitive components is found in the memory (average result of 64.5% out of 100%) and attention functioning (average result of 67.4% out of 100%) components. It is understandable that the MMSE scores are close to the normal cognitive functioning as it is a nonspecific screening test for global cognitive assessment. The results are shown in percentage of the corresponding score meanings of each scale. The normal values of the scores for the attention and memory tests are equal to >84% normal functioning, 84–68% mild decline, 68–58% moderate decline, and <58% severe decline. The MMSE scores are—>84% norm and 84–78% borderline with mild cognitive deficit. Aphasia scores range from 100–84% norm, 84–75% mild aphasia, and <75% moderate aphasia.

All of the patients were provided with recommendations to follow a hygienic-dietary regime and increase physical and social activity. Neuroprotective agents such as Citicoline/600–1000 mg/d, Piracetam/up to 2400 mg/d and Memantine/up to 20 mg/d were prescribed to the patients with evident cortical atrophy and the agent was chosen individually after a consultation with neurologist.

**Figure 2:**
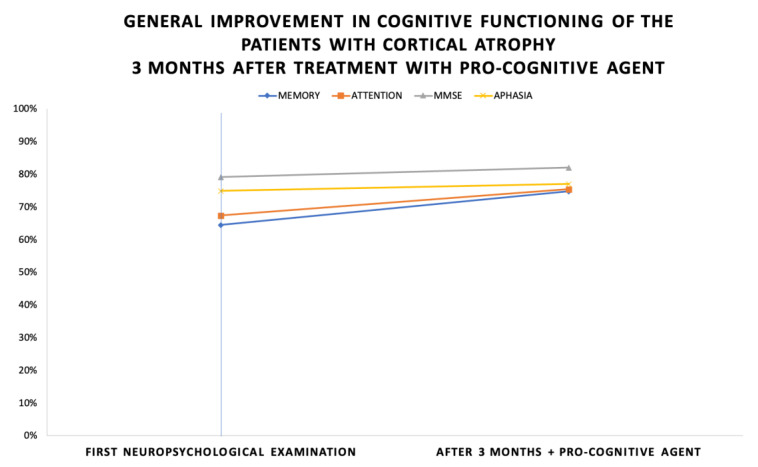
General improvement in cognitive functioning of the patients with cortical atrophy 3 months after treatment with pro-cognitive agent.

After a follow-up period of 3 months, positive results of the neuropsychological examination were reported. The most significant improvement of cognitive functioning is shown in the memory scores—10% (average scores of 65% in the initial testing and 75% after 3 months). Attention functioning improves by 9% (average scores of 67% in the initial testing and 76% after 3 months). MMSE improves by 3% (average scores of 79% in the initial testing and 82% after 3 months). The Boston Naming test for aphasia improves by 2% (average scores of 79% in the initial testing and 82% after 3 months). Figure 2 shows these dynamics.

The second group consisted of 16 patients who were aged between 25 to 50 years (with mean age 38 SD 4.8) Fifty six percent (nine) of the group were females, the rest (seven) were males, 31% were married and 50% had a university degree. Those patients were diagnosed with anxiety and personality disorders as well as with somatization symptoms in addition to the cognitive impairment. The anxiety and somatization features were associated with the COVID-19 infection and the patients did not have previous history of psychiatric disorder. One of the patients had features of borderline personality disorder, which is a condition that does not affect general cognition.

These patients had normal imaging findings. The results of the neuropsychological examinations in the group indicate a slight decrease in fixation and memory retention (average scores of 77% out of 100%). A decrease in the range, concentration, distribution and switching of attention was found (average scores of 82% out of 100%). The MMSE results for general cognitive assessment indicated normal scores (average scores of 94% out of 100%) and the Boston Naming test’s results for aphasia (average scores 88% out of 100%) were within the norm (but slightly prolonged latent time for answering was observed).

**Figure 3:**
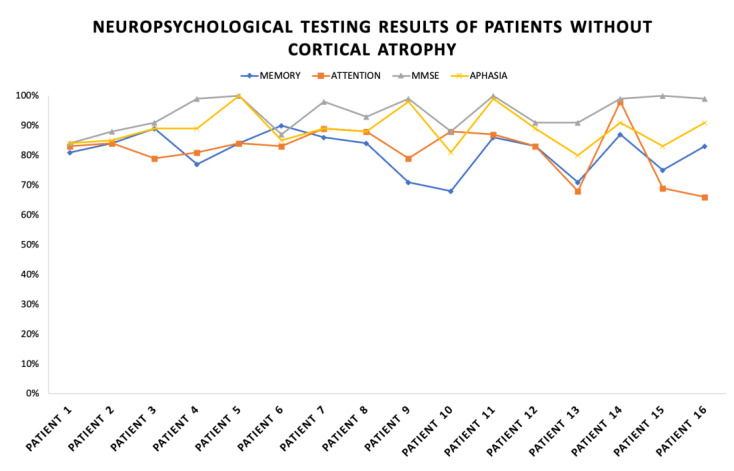
Neuropsychological testing results of patients without cortical atrophy.

Figure 3 shows the results from the neuropsychological examination of each patient. Overall, better cognitive functioning is observed compared to the previous group. Similarly, the largest decline in the cognitive components is found in the memory and attention functioning.

These patients were also provided with recommendations to follow a hygienic-dietary regime, increasing physical and social activity, but no medication was added to improve their cognitive functions. After 3 months, a positive change was reported during a secondary neuropsychological examination and according to their subjective assessment as well.

**Figure 4:**
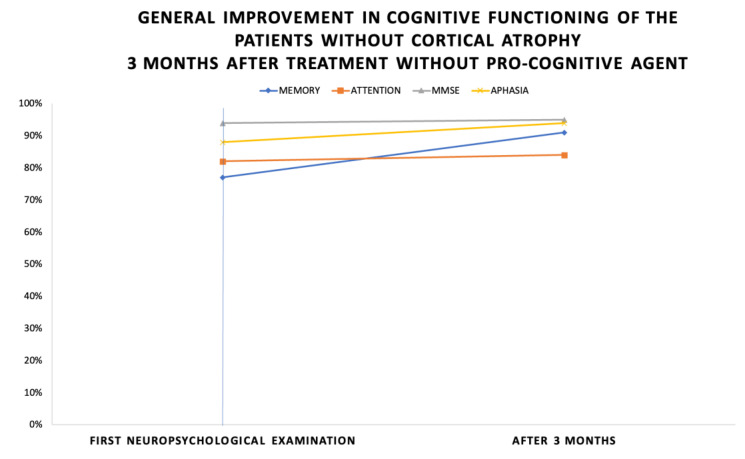
General improvement in cognitive functioning of the patients without cortical atrophy 3 months after treatment without pro-cognitive agent.

The results of the improvement of cognitive assessment of the second group are shown on Figure 4. The most significant improvement of the cognitive functioning was found in memory—14% (average scores of 77% in the initial testing and 91% after 3 months). The improvement was 4% more than in the previous group. This might be a result of the better recovery potential of the patients without evident brain pathology. Attention functioning improved by 2% (average scores of 82% in the initial testing and 84% after 3 months). MMSE improved by 1% (average scores of 94% in the initial testing and 95% after 3 months). The Boston Naming test for aphasia interestingly improved by 6% (average scores of 88% in the initial testing and 94% after 3 months).

**Figure 5:**
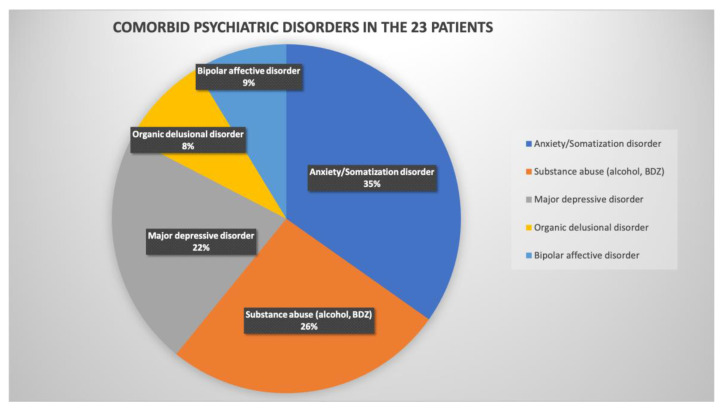
Comorbid psychiatric disorders in the 23 patients with objective cognitive decline in the course of post-COVID syndrome.

Figure 5 shows the prevalence of different comorbid psychiatric diagnoses in all of the 23 patients who appeared to be with objective cognitive decline in the course of post-COVID syndrome. The most common diagnosis in these patients was anxiety/somatization disorder (35%), followed by substance addiction syndrome (26%) and major depressive disorder (22%), which adds to the epidemiology data of psychiatric complications in the post-COVID period, published by [28,29,30].

**Figure 6:**
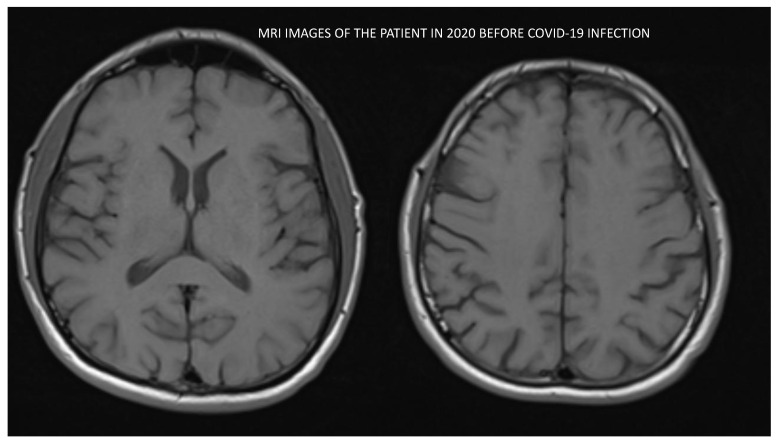
MRI image of the patient conducted in 2020 before the COVID-19 infection.

**Figure 7:**
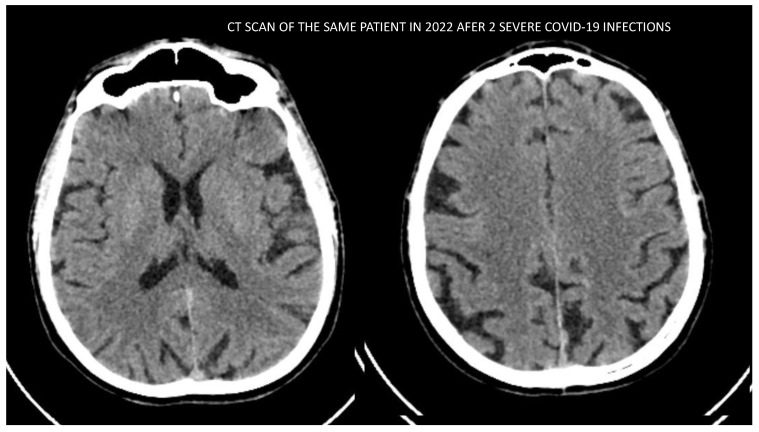
CT scan image of the patient conducted in 2022 after surviving 2 severe COVID-19 infections.

Finally, we present the neuroimaging examinations of one of the patients from the group with evidence of cortical atrophy. His imaging findings are the most distinguishing and demonstrative and show explicitly the damaging effect due to severe COVID-19 infection on the brain structures. The patient had no history of any previous infection cognitive complaints; he did not suffer from any neurodegenerative disease nor had any history of hypertonia, obesity or any family history of dementia or psychiatric disorders. According to the anamnestic data from his family, he led a healthy lifestyle, had a BMI in the normal range, was nonsmoker and had no history of alcohol abuse/addiction. At the time of our investigation, he was 58 years old. He had a history of having the COVID-19 infection (in 2021 and 2022) twice with two hospitalizations and pulmonary complications, need for oxygen therapy (up to 10 L/min) and high doses of corticoids (up to 8 mg Dexamethasone per day). The first image (Figure 6) was made in 2020 before the COVID-19 infection. The second image (Figure 7) was made after the acute phase of the second COVID-19 infection. We see significant progression in the severity of the cortical atrophy. In addition to the cognitive decline, this patient presented with organic delusional disorder—mostly expressed by delusions on paranoid themes and later the psychiatric clinical picture changed to nihilistic delusions, which are more typical for depression with psychotic features. Through this period, the general functioning of the patient was dramatically impaired.

## 4. Discussion

The pathophysiological mechanisms by which SARS-CoV-2 affects organs are multimodal. However, the exact processes leading to the neurological and psychiatric consequences of COVID-19 are still not fully understood. The following neurotoxic mechanisms have been described in coronavirus infections:

1. Neurotropism and direct ability to infect neurons and glial cells. The consequences of these processes lead to neuronal dysfunction and damage (neuroinvasion). The virus can reach the central nervous system (CNS) indirectly through the blood–brain barrier and/or directly by axonal retrograde transport through olfactory nerve neurons [31,32].
2. Affecting cerebral blood vessels and cerebral microcirculation (coagulopathies) through ACE2-R adhesion, leading to ischemic or hemorrhagic strokes [33,34].
3. Secondary negative consequences of excessive systemic inflammatory reactions, the so-called “cytokine storm” and peripheral organ dysfunction affecting the brain [35].
4. Global ischemia due to respiratory failure, oxygen therapy and mechanical ventilation, as well as the so-called acute respiratory distress syndrome (ARDS) [36,37].
5. One additional hypothesis claims that SARS-CoV-2 infects endothelial cells of the blood brain barrier which allows direct transport in the CNS by causing viremia [37,38].

Taking into consideration all the data provided, we can conclude that SARS-CoV-2 is a potentially dangerous neurotropic pathogen and therefore COVID-19 infection increases the risk of developing neurological and neuropsychiatric complications.

Due to the multifactorial pathophysiological mechanisms by which SARS-CoV-2 affects the organs, we summarize that after the clinical observation, together with the specific neuropsychological tests and neuroimaging examinations, in 23 patients out of 120 (19.17%) with a history of acute COVID-19 infection, cognitive decline was found, more particularly in fixation and retention memory, concentration and sustained attention, and mild to moderate aphasia. Our results correspond with the already published data on the prevalence of cognitive disturbances in patients who had COVID-19 infection [18].

The conclusions we can elicit are preliminary due to the small size of the studied sample; however they show a disturbing tendency of cognitive impairments in the post-COVID period in the age group up to 50 years with a lack of neuroimaging brain changes. Our study indicates that in each case of subjective cognitive complaints, it is mandatory to conduct a neuropsychological examination to objectify the condition and to imply certain prophylactic measures in order to prevent its worsening.

Moreover, our experience shows that after implementation of a hygienic-dietary regime, including increased physical and social activity, without taking specific pro-cognitive medications, after 3 months a positive change was reported in the secondary neuropsychological examination as well as in the subjective report of the subjects.

We assume that the patients with evident cortical atrophy most likely had discrete asymptomatic cognitive decline before infection with COVID-19. These patients were also recommended to follow hygienic-dietary regimen, including increased physical and social activity. Additionally, a neuroprotective pro-cognitive medication was included in their treatment for a period of 3 months. The chosen agents (Citicoline/600–1000 mg/d, Piracetam/up to 2400 mg/d and Memantine/up to 20 mg/d) are well-established and highly used in the neurological and psychiatric practice in Bulgaria for patients with cognitive impairment of any kind. They were prescribed individually after consultation with a neurologist.

Citicoline is a well-tolerated neuroprotective agent, promising to improve cognitive disturbances more likely of vascular origin. It has a proven membrane stabilizing effect on neuronal brain cells with ischemic damage [39,40]. Recent studies show its potential role as adjunctive therapy in COVID-19 related cognitive decline [41,42].

Piramem, although an old and well-known drug with controversial efficacy, has been proven to improve cell metabolism and enhance neuroplasticity in the elderly patients with vascular brain pathology [43,44,45,46]. In a controlled clinical trial of psychiatric patients with mild cerebral impairment, piracetam treatment improved overall functioning, particularly alertness, socialization, and cooperation, relative to the control group [47].

Memantine is another agent, with NMDA weak/noncompetitive/antagonist properties, which prevents neuronal cell death by decreasing excitotoxicity (a result of NMDA-R overactivation) [48,49]. Although its primary target is aimed at patients with Alzheimer’s disease, research showed that a dose of the agent of 20 mg/day might be efficient for moderate to severe Alzheimer’s disease and for patients with severe demented syndrome, as well as cases of vascular dementia [50,51,52].

After 3 months, the patients shared their subjective feelings of improvement and after conducting a new neuropsychological examination, a positive change in the objective results was reported.

Although there are limited data on the benefit of prescribing pro-cognitive agents in the post-COVID period, our clinical experience suggests that it might be a useful part in the process of recovering from the infection’s consequences on cognition for patients with brain pathology.

Limitations of the study are the small size of the sample, the lack of randomization and more strict control of the neuroprotective treatment, comorbidity with psychiatric disorders and the short period of following the patients’ results. However, studies like ours might be a foundation for deeper investigations in the field of improving cognition complications as a result of viral infections and inspire more strictly controlled trials of use of neurotropic agents in patients with cognitive deficit associated with COVID-19 infection.

## 5. Conclusions

Patients with post-COVID syndrome are at high risk of experiencing global cognitive impairment to a large extent which manifests mostly with a decrease in attention and memory functioning. Thorough neuropsychological and neuroimaging examinations need to be conducted in each case of subjective complaint of cognitive impairment. Overall, more detailed prospective and controlled neuropsychological and neuroimaging studies would be of great benefit to provide more information about the treatment of cognitive disturbances associated with the post-COVID syndrome and the rehabilitation of these patients.

## Data Availability

Not applicable.
